# Towards fair and effective North–South collaboration: realising a programme for demand-driven and locally led research

**DOI:** 10.1186/s12961-017-0251-3

**Published:** 2017-11-13

**Authors:** Maarten Olivier Kok, John Owusu Gyapong, Ivan Wolffers, David Ofori-Adjei, Elis Joost Ruitenberg

**Affiliations:** 1 Institute of Health Policy and Management, Erasmus University Rotterdam, Burgemeester Oudlaan 50, 3062 PA Rotterdam, The Netherlands; 2 Amsterdam Public Health, VU University Amsterdam, Amsterdam, The Netherlands; 3 Institute of Health Research, University of Health and Allied Sciences, Ho, Ghana; 4 Department of Health Care and Culture, VU University Medical Centre Amsterdam, Amsterdam, The Netherlands; 5 Noguchi Memorial Institute for Medical Research, University of Ghana, Accra, Ghana

**Keywords:** Health research system, North–South collaboration, Capacity strengthening, Multilevel, Contribution mapping

## Abstract

**Background:**

At the turn of the 90s, studies showed that health research contributed little to health and development in low- and middle-income countries because it was oriented towards international priorities and dominated by researchers from the North. A new approach to North–South collaboration was required that would support demand-driven and locally led research in the South. The aim of this study was to analyse the development and functioning of a programme for demand-driven and locally led research in Ghana that was supported by a North–South collaboration.

**Methods:**

For this mixed-method case study, we combined document analysis, key informant interviews and observation of programme events.

**Results:**

The development of the research programme started with constructing a sponsorship constellation in the Netherlands. After highlighting the problems with traditional research collaboration, an advisory council formulated a vision for a more equal and effective approach to North–South collaboration. Together with Ghanaian partners, this vision was turned into a proposal for a Ghanaian-led programme for demand-driven and locally led research, which was funded by the Netherlands government. Research priority setting showed that the Ghanaian research needs were very different from the priorities of foreign funders and researchers. After a slow start, the number of locally submitted proposals increased from 13 in 2001 to 94 in 2005, revealing the existence of a substantial, but partly latent reservoir of research capacity. In total, 79 studies were funded. An impact evaluation showed that the results of the majority of the studies were used to contribute to action. Despite its success, the research programme came to an end in 2008 after the sponsorship constellation in the Netherlands fell apart.

**Conclusion:**

Our study shows that realising a programme for demand-driven and locally led research in the South provides an effective approach to North–South collaboration in which results are used and local capacities and institutions are strengthened.

## Background

For decades, researchers, funders, policymakers and other stakeholders have searched for better ways to organise research to contribute to health. The publication of the report of the Commission on Health Research for Development in 1990 signified an important change in thinking about research for health in low-income countries [1]. The report stated that conventional health research did not match with the priorities in the global South and that existing North–South research collaboration was often counterproductive. The report described an enormous global mismatch between health needs and research investments, which was later referred to as the 10/90 gap, where less than 10% of the global research investments was oriented towards 90% of the global health burden. Yet, the global mismatch between health needs and research investments was only one part of the problem.

Analyses showed that existing North–South research collaboration could constrain development by disturbing research priorities in the South and rewarding those who went along [2]. Researchers, donors, and governments from the North had their own priorities and interest in the South, which strongly influenced what was being studied and how. The North’s focus on universally applicable, biomedical insights and technological solutions and scientific publications as a measure of excellence could hamper the emergence of national research systems in the South by orienting talented local researchers to international agendas, instead of local needs and societal relevance [3]. This could fuel a vicious cycle in which local authorities did not engage with research because it did not fit their needs, and Southern researchers became internationally focussed and locally isolated because of a lack of local investment. Despite the good intentions, international research collaboration could constrain development.

One response to the 1990 report was to invest more in researching diagnostics and treatments for diseases that had been neglected globally [4]. Prioritising this research seemed wise because the outputs were expected to be universally applicable. While promising, this ‘globally oriented’ research provided only a fraction of the knowledge required for improving health in low-income countries [1, 5]. At least as important was the locally specific research that countries needed in order to improve health outcomes and equity in their own situation [1, 6]. This research had to be oriented towards local demands and was best conducted by researchers who understood the local circumstances, interacted with intended users and could assist in translating results into action [3, 7, 8]. In this article, we focus on the development and functioning of a programme for such demand-driven and locally embedded health research in a low-income country.

In the early 90s, the Netherlands government took the initiative to develop a new type of research collaboration that would combine demand-driven and locally led research with a genuine and equal North–South partnership. The Ghanaian government was interested in developing such a collaboration because it was trying to make health research more relevant for national development. Together with their Dutch partners, they designed an inclusive research programme in which different voices in Ghanaian society were engaged in setting a national research agenda [9]. Each year, Ghanaian professionals were invited to submit research proposals that matched this priority agenda. While Ghanaians led the studies, they could invite Dutch researchers to participate as co-investigators. After a long preparation, the Ghanaian-Dutch Health Research for Development Programme (HRDP) commenced in 2001 and funded a total of 79 locally led studies that were oriented towards the national research agenda.

The HRDP was presented as a new type of approach to North–South collaboration in health research for development [9]. Initiatives for strengthening research capacity typically focus on training individual researchers and strengthening the directly involved institutions [6, 10–12]. In addition, there are initiatives that assist countries in developing and strengthening a research system by, for instance, assisting with formulating research policies and setting research priorities [13–17]. While individual, institutional and system capacities all seem important, the effects of capacity strengthening tend to be constrained by a lack of funding for demand-driven research [6, 18–20]. In most low-income countries, the research funding provided by the government is barely sufficient for maintaining a basic research infrastructure and paying the salaries of local researchers. Meanwhile, international research funders continue to push their own priorities, instead of aligning with national research agendas [6, 18, 21]. Given these challenges, the approach of the HRDP provides a promising alternative. Instead of focusing on individuals, institutions or systems, the HRDP set out to realise an actual programme for demand-driven and locally led research, embedded in a low-income country and supported by a North–South partnership. The aim of this study is to analyse how this programme for demand-driven and locally led research came into being and functioned in practice.

### Analytical framework

To guide our study of how this demand-driven research programme came into being and functioned in practice, we use a multilevel framework that builds upon existing literature on the functioning of scientific research. In most analyses of research capacity strengthening, a distinction is made between phenomena at three different levels that are required for the functioning of research, i.e. individual researchers, the institutions in which they function and the macro-level context [5–8, 19, 22–24]. A similar distinction between three different levels is made by scholars who study the functioning of modern science in industrialised countries [25, 26]. Our analytical framework is based on the idea that phenomena at three different levels are required for the functioning of health research.

The first level in our model concerns the actual research on location. This encompasses all that is directly involved in producing research knowledge, including competent researchers, those who support them, the social and physical space in which they produce knowledge and all that is required for collecting observations, analysing data, and designing and disseminating transferable knowledge claims.

The second level concerns the set of dedicated institutions and networks that is required for demand-driven and nationally embedded health research. This includes the actors, organisational structures, practices and all types of resources involved in setting a national research agenda, generating and selecting research proposals, funding and supporting research projects, and disseminating results. In addition to being a platform for engaging societal stakeholders, this second level also includes networks for interaction within the research community and the infrastructure that enables this.

The literature about research capacity strengthening is the least clear about the nature and role of the third level. Different actors and structures, such as governments [27], donors [24], the profile of research in the media and amongst policymakers and citizens [23], legal frameworks [23], international agencies [8], donor funding [19], national demand for research, political will and colonial histories [28], are described as being part of this third level. Some authors refer to this third level as something external, using terms such as context [19], the external environment [23] or the macro-level context [6]. At the same time, authors point to the influence that these actors and structures have on the research that is being conducted, which suggests that this third level is not external, but an integral part of the functioning of research [5, 6, 23, 27, 28].

**Fig. 1:**
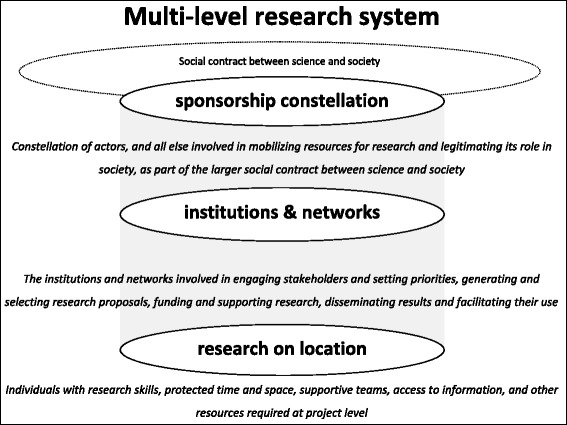
Multilevel research system

Analyses from the field of science studies help to specify the nature and role of this third level. Historical and sociological studies show how, within modern science, research on location and the required institutions and networks are functionally dependent upon a third constellation of actors, ideas and structures that fulfils two core functions, namely mobilising resources for research and legitimising its role in society. This constellation is at the core of what Guston describes as the ‘social contract’ between science and society [26, 29]. Because our study focusses on a specific research programme, we will not assess this larger social contract, but rather the specific sponsorship constellation that mobilises resources for a research programme and legitimises its role in society. Our analytical framework, representing these three different levels, is illustrated in Fig. 1.

## Methods

For this in-depth case study, data were collected between 2005 and 2012 through document analysis, key informant interviews and observation of programme events.

### Organisation of data collection

The documents used for this analysis are reports from the Development Assistance Research Council (RAWOO), the 2001 Programme of Work of the HRDP, a book chapter about the design of the HRDP, health policy documents from the Ministry of Health and the Ghana Health Service, yearly reports of the Health Research Unit of the Ghana Health Service, research proposals and final reports of the research projects, and minutes and reports of programme meetings and workshops. We also analysed the report of a review of the HRDP that was conducted in March–April 2005 by two Ghanaian consultants.

The second method of data collection was interviewing purposively selected key informants. The majority of the interviews were conducted for a detailed impact evaluation of the individual studies, which is published elsewhere [30]. For this impact evaluation, 113 interviews were held during four periods between May 2005 and June 2011. These interviews focussed on the contribution to action of the first 30 research projects that were funded by the HRDP. This impact study was led by the first author of the present study, who was supported by three other external researchers. The interviews for this impact analysis focussed on how these 30 research projects were formulated, how they evolved over time and how the results were used to contribute to action. For each case that was assessed, the principal investigator and/or co-investigators and potential key users were interviewed, amongst many others [31]. For these interviews, a combination of a questionnaire and a topic list was used. The investigators who were interviewed for this study were asked about issues such as their previous experience with research and health policy, reasons for being involved in research, collaboration with researchers from the North, career perspectives, constraints in the research environment and their perspective on the functioning of the research programme.

In addition to the interviews for the impact evaluation, we conducted 16 interviews with 11 purposively selected key informants who were directly involved in the development, daily management and termination of the programme, such as research coordinators, programme management, members of the Joint Programme Committee and staff of the Royal Netherlands Embassy. Notes were taken during all interviews and seven interviews were recorded and transcribed. Based on notes and/or the transcription, a detailed summary was made of each interview.

The third method of data collection was the observation of events that were organised by the HRDP, such as capacity-building workshops, seminars at which research was presented and meetings at which the functioning, continuation and termination of the HRDP was discussed, in both Ghana and the Netherlands. Notes were made of these observations.

One interviewer and a data management assistant were involved in data analyses. Data were analysed manually by identifying and coding statements in documents, notes from observations and interview summaries according to topic. This was done together by the lead researcher and a data management assistant, after which the emerging themes were discussed. Summaries were then made for each theme using a constant comparative method of analysis [32]. Our analysis was recursive, constantly moving from specific examples and events to the more general chronological description, with the aim of identifying the most relevant dynamics and patterns.

Using these theme-specific summaries, a thick description was drafted of the three chronological phases of the research programme (the development phase until 2001, the functioning between 2001 and 2006, and the ending of the programme). This process description was used to draft a first version of this article, which was shared with two Dutch and two Ghanaian members of the Joint Programme Committee who had been involved in the development and operations of the research programme from the early 90s until after its formal ending in 2008.

This study did not require ethics approval in Ghana. Under Dutch law, ethics approval in the Netherlands was also not required. Even though formal approval was not required, we followed normal ethically responsible qualitative research practice to ensure that substantive ethical issues would be dealt with appropriately. Informed consent to participate in the study, record the interviews, use quotations and publish the results was obtained from all study participants. A report with the preliminary results was shared with participants in 2008. The preliminary results were presented and discussed at a meeting with participants in Ghana in 2008 and at a meeting in the Netherlands in 2009. Those involved in the discussions confirmed the presented results.

## Results

We present the results in three parts. We start by showing how, in the early 90s in the Netherlands, a vision for a new approach to North–South research collaboration was developed, which, together with Ghanaian policymakers, researchers, and health sector and NGO representatives, was turned into a proposal for a programme for demand-driven and locally led research in Ghana. In the second part, we focus on the functioning of the research programme and the efforts and dynamics involved in increasing its performance. In the third and final part, we show how the research programme came to an end after changes in development policy led to the collapse of its sponsorship constellation in the Netherlands.

### 1990–2001: translating a vision into a research programme

The development of the HRDP started in the early 90s in the Netherlands. At the time, numerous scholars from Dutch universities were involved in health research in low-income countries. Most of this research focussed on specific diseases such as malaria, tuberculosis and leprosy. This research was mainly funded through the Science Councils of the Netherlands Organisation for Scientific Research (NWO), which was funded by the Ministry of Education, Culture and Science. The NWO Science Councils represented the interest of Dutch academia and focussed on scientific excellence, which was described as publishing new insights in leading academic journals.

#### Problematising existing research collaboration

The origin of the new approach to North–South collaboration can be traced back to the late 80s, when the success of development aid, including the contribution of health research to development, was problematised [33]. In 1990, the newly appointed Minister for Development Cooperation in the Netherlands asked the RAWOO to study problems with existing research collaboration and provide advice on how the focus of health research for development could be geared more towards the needs of the South. In several reports, the RAWOO laid out why traditional research collaboration contributed little to health and development in low-income countries [34]. The main problems were that research for health in the South was mostly driven by the priorities of funders in the North, matched poorly with local needs and had a narrow focus on specific diseases. Research was mostly initiated and led by foreign researchers, there was little funding for locally specific, social and health systems research and there was little attention for the local dissemination and use of results [34, 35]. Within the South, research was often geared towards the interest of the elite, instead of the more marginalised. Due to the dependence on external funding, local research talent had to focus on international priorities and was turned away from national needs and local networks (RAWOO 1996). North–South research cooperation had helped to train researchers in the South, but had contributed little to the development of national institutions that were required for demand-driven and locally led research.

In response to these problems, the RAWOO formulated a vision in which health research for development should be (1) demand-driven, geared towards national priorities of countries in the South; (2) participatory, including all stakeholders in the South, especially the more marginalised; (3) strengthen local capacities of individuals, networks and institutions; (4) societal, multi-disciplinary research was required to deal with issues such as health; and (5) context specific, i.e. to be applicable, knowledge had to relate to local circumstances.

The Netherlands Minister for Development Cooperation supported this new vision and asked the RAWOO to collaborate with the NWO Science Councils to jointly translate these ideas into a new type of research programme that would generate demand-driven, locally led and nationally embedded research for health in a low-income country.

#### Struggle during the preparatory stage

The translation of this new vision into an actual research programme resulted in a long struggle in the Netherlands between the Science Councils and the development-oriented RAWOO. In June 1995, the RAWOO proposed a four-step process to develop a research programme together with a partner country in the South. The steps were to (1) identify and shortlist potential partner countries in the South; (2) map the health research situation and potential for collaboration in selected countries; (3) set up a local Steering Committee for a priority-setting process in the selected country; and (4) based on this country-specific research agenda, invite Dutch researchers to jointly develop a plan that would result in a programme for demand-driven and locally embedded research.

To facilitate the programming process in the Netherlands, a Programme Study Committee was set up with representatives of the RAWOO, the Science Councils and other stakeholders. At the first meeting of this new committee, representatives from the Science Councils started to question the approach that was proposed by the RAWOO. Science Council members claimed that a new programme should focus on scientific excellence and argued that engaging local stakeholders in the South would be very complicated. Instead of asking stakeholders in the South about their needs for research, Science Council representatives proposed that research priorities should be identified in the Netherlands before selecting a partner country in the South. The RAWOO members defended their ideas for a new approach by arguing that research for development should be demand-driven, locally led and embedded within a national infrastructure that would facilitate its use. Since health policies were mostly made within national systems, health research for development should be embedded in national structures, and not just be linked to a theme [2].

#### Selecting a partner country

While the discussions in the Netherlands between the Science Councils and RAWOO were ongoing, a partner country in the South had to be selected. To protect their existing research collaborations, Science Council members insisted that the new programme should start in a country in which they were not very active. After extensive consultations, Mozambique, Benin and Ghana were selected as potential partner countries. From March to April 1996, a Dutch research team interviewed 59 participants in these three countries to map the present state of health research, ongoing research activities, capacity-building needs and the potential for collaboration [2].

The mapping study provided further evidence for the problems with existing research collaboration and showed the need for a demand-driven approach. The report of the mapping study provides some illustrative quotations [3]. The Vice-Minister of Health in Mozambique confirmed the influence of the North on the research agenda: “*Research is influenced by donors’ fashion, donors’ interest. We are heavily dependent on donors*”. Another informant pointed to the consequences of the lack of local funding: “*Each institute is developing towards isolation. We have not enough State funding*”. Others addressed the difficulties with accessing scientific articles, and pointed to the mutual dependency that reproduced the existing system: “*It is a kind of trade: they need the field, we are getting some funding*”.

Based on the mapping study, Ghana was invited to jointly develop a new research programme. The Dutch were eager to collaborate with the Ghanaians because they had met with enthusiastic research advocates at the Ghanaian Ministry of Health, who aimed to make health research more useful for national development and had decentralised research to three health research units that were located in the north, centre and south of the country, with a coordinating research unit in the capital Accra [36]. While the Ghanaian government funded this research infrastructure, it provided no significant funding for demand-driven and locally led research. Local researchers generally depended on foreign funders, collaboration with foreign partners and international research priorities, and were keen to initiate and lead their own studies.

While the Ghanaians were invited to collaborate, the struggle between the Science Councils and the RAWOO continued. Science Councils representatives tried to change the way the programme was developed by suggesting that the Ghanaian research priorities should be taken as starting point for developing a thematic programme for the region. Next, they proposed to restrict priority setting in Ghana to areas in which Dutch researchers had considerable expertise. Difficult negotiations and strong support from the Netherlands Minister of Development Cooperation were required to continue the preparatory process.

#### Agenda-setting workshop in Ghana

In August 1996, a local steering committee was set up in Ghana, which was tasked with organising an agenda-setting workshop. Three groups of research stakeholders, which were referred to as ‘the three voices’, were identified to be engaged in the process. These were (1) health policymakers at all levels, (2) the research community, and (3) end-users, including health workers and NGO representatives who would serve as proxies for the more marginalised in Ghanaian society.

In March 1997, the first agenda setting workshop was held in Ghana. Over 100 participants from the government, health sector, research community and NGOs gathered for the first time to discuss the research needs of the Ghanaian health sector. A Ghanaian researcher later recalled the meeting and the diversity of participants: “*everyone was together, from the ministry, policy, from research, many, you know, from NGOs* […] *I was surprised to see the people who were at the meeting. Some of them were from very grass root organisations who are operating small projects in the Volta Region and decided to come out.* […] *It was demystifying research as something that just academics do*”.

The workshop resulted in a list of principles for a research programme within a North–South Collaboration:

1. The research agenda should be based on national needs
2. Ghanaians should take the lead in research projects and in choosing partners
3. Research should be inter-disciplinary and engage stakeholders throughout the process
4. Research should be integrated with capacity-building
5. Research cooperation should be based on mutual respect

#### Programme development workshop in the Netherlands

The next step in the development of the programme was to organise a workshop in the Netherlands to discuss how the Dutch research community could contribute to the Ghanaian research needs. The Dutch researchers were keen to draw up a list of research topics that they could focus on. The Ghanaian representatives were more interested in how the programme would be organised and emphasised that agenda setting should be an ongoing process that would be driven by local needs. In the end, participants recommended to set up a Joint Programme Committee with Ghanaian and Dutch representatives who would guide the development of the programme and the priority-setting process.

#### Drawing up the final programme

In the Netherlands, the struggle between the Science Councils and RAWOO continued until the final programme proposal was submitted to the Minister in November 1997. For several months, representatives of the Science Councils refused to support and sign the programme proposal. They argued that the Dutch researchers had not been treated as equals during the design of the proposal and insisted that the research programme should be based on themes instead of national priorities and scientific quality criteria instead of societal needs. They demanded a Dutch steering committee that could overrule the Joint-Programme Committee. At its last meeting, the Programme Study Committee could not agree on how the programme should be led and asked the minister to decide.

In February 1998, the minister decided to fund a pre-implementation stage in which the Joint Programme Committee could set up and oversee task forces that would develop a first research priority agenda, identify capacity-building needs, draw up a strategy for enhancing research use and design an organisational structure. Soon after, elections were held in the Netherlands and a new minister for Development cooperation was installed, which delayed the process on the Dutch side. In 1999, the results from the taskforces were brought together and a 5-Year Programme of Work was drafted, which was submitted to the new Dutch Minister for Development Cooperation, who approved it at the start of 2001.

#### The Ghanaian-Dutch health research for development programme

The 5-Year Programme of Work describes the strategy of the research programme, its organisational structure and the expected outputs. It stated that the research programme had three pillars, namely (1) to better attune health research to the needs of the public policymakers and end-users or beneficiaries in Ghanaian society at large, thus making it more demand-driven; (2) to put greater emphasis on the need to strengthen national capacity for health research, and to enhance local ownership by empowering the Ghanaian research partners and local stakeholders; and (3) to redress imbalances in North–South collaborative research by promoting genuine research cooperation between Dutch and Ghanaian researchers, which should be based on mutual trust, joint learning and equal say, and influence in decision-making and programme management.

#### Organisational structure of the HRDP

**Fig. 2:**
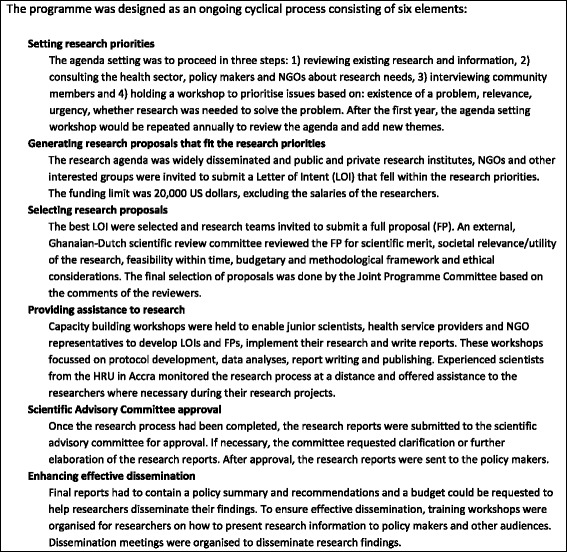
Programme cycle of the HRDP

The research programme followed a demand-driven programme cycle (Fig. 2). The research programme was managed by the existing Health Research Unit of the Ghana Health Service in Accra and formally led by the Joint Programme Committee, which was made up of three Ghanaian and three Dutch members. A separate secretariat in the Netherlands facilitated the process of involving Dutch researchers and would fund their work from a separate budget. For the first 5 years of the research programme, US$3.4 million was made available by the Netherlands Minister of Development Cooperation.

In this first part, we trace the 10-year preparatory process in which a new vision for North–South collaboration was developed and translated into a research programme for which a sponsorship constellation was established. The development of this programme started with problematising the contribution of research to health and development in the South and the traditional power relations that favoured the interest of researchers from the North. This problematisation inspired a new Minister for Development Cooperation in the Netherlands, who asked an expert committee to develop a vision for a more equal and effective approach to North–South research collaboration. Meanwhile, in Ghana, engaged policymakers were decentralising health research to three units and were aiming to orient research to the needs of the health sector. While Ghanaian and Dutch representatives set out to develop a more equal and effective research programme, representatives from science organisations in the Netherlands opposed the plan to use development funding for research that would focus on Southern needs and would be led by Southern researchers. A series of meetings and a thorough priority-setting process in Ghana confirmed the need for a demand-driven and locally led approach and was essential for developing the programme proposal that was eventually funded in 2001. The decision to approve and fund the 5-Year Programme of Work stabilised the sponsorship constellation in the Netherlands and allowed the demand-driven research programme in Ghana to start.

### 2001–2006: making a demand-driven research programme work

In this second part, we describe how the research programme functioned during the 5 years in which it was fully operational and funded 79 locally led studies in Ghana.

#### Research priority setting

The priority setting process showed there was a true need for demand-driven research in Ghana (Box 1). The national research agenda was very different from the priorities of foreign researchers and international funders. The four-page priority agenda did not mention any specific diseases, which used to be the main focus of research driven by the North. Besides different themes, participants in the agenda-setting process also emphasised the need for locally specific research. Examples include health beliefs among Ghanaians, reasons for enrolling in health insurance, local problems with antimicrobial resistance and differences in prices between the public and the private sector.

**Box 1** The four themes and topics of the research agenda

**Table:** 

1)	Communication and community participation Specific needs: health education approaches in Ghana, beliefs relating to health and prevention, evaluation of existing communication approaches and related interventions in the field of the Priority Health Service Interventions, piloting community involvement in policy formulation, planning, implementation and evaluation at district level, and institutionalising community involvement.
2)	Quality of healthcare Specific needs: staff attitude, referral system, assurance of technical skills of providers, drugs and logistics management, and monitoring and confronting antimicrobial resistance.
3)	Financing of healthcare Specific needs: managing internally generated funds, improving management, formal and informal charges, pricing of drugs and services, introducing standardised pricing, comparative prices in private and public sectors, exemptions, especially for the poorest and most vulnerable, and cultural- and gender-sensitive mechanisms to target the truly indigent and most vulnerable clients.
4)	Decentralisation of healthcare Specific needs: multi-sector coordination, integrating funding and balancing national and local priorities.

Participants were positive about the diversity of stakeholders that participated in the agenda-setting process. Policymakers had lobbied for issues related to health financing, decentralisation and quality of care that lay at the core of the 2001–2006 Health Sector Programme of Work of the Ghana Health Service. The academic community advocated for more biomedical issues, such as the status of antimicrobial resistance, and NGO representatives emphasised themes such as community engagement and access to care for the most vulnerable, poorest of the poor and truly indigent. Participants reported that, besides articulating priorities, the agenda-setting process was also useful for learning about ongoing research and policy processes and building a diverse national network of people engaged with demand-driven research.

#### Generating and selecting research proposals

In the first years of the programme, it proved more difficult than expected to generate locally led research proposals. In response to the first call for proposals in 2001, only 13 Letters of Intent were submitted and their methodological quality was below expectations. The disappointing number and quality of the proposals raised questions about whether sufficient local research capacity existed. The Joint Programme Committee insisted on keeping up its scientific standards and invited only six research teams to submit a full research proposal, of which it considered five good enough to be funded.

**Fig. 3:**
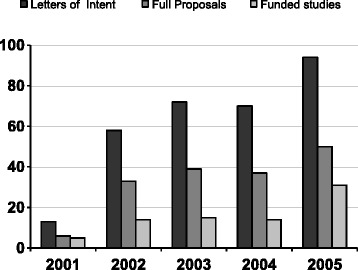
The number of submitted letters of intent, full proposals and funded studies

In response to the disappointing start, the programme secretariat tried in different ways to increase the number and quality of the proposals that were submitted. To reach more potential applicants, the secretariat advertised the second call for proposals in two national newspapers and promoted the programme in professional networks and during health sector meetings. To improve the quality of the proposals, the secretariat organised workshops in which applicants with good ideas could learn how to write a robust research proposal. In the subsequent years, both the number and quality of the letters of intent and full research proposals improved substantially, with 94 Letters of Intent and 31 funded studies in 2005 (Fig. 3).

**Fig. 4:**
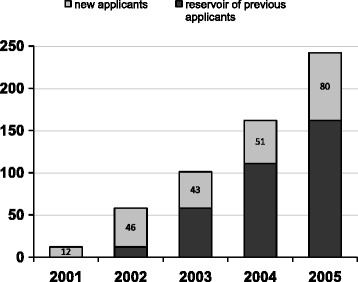
The number of new applicants that submitted a proposal per year

The rapid growth of the number and quality of the research proposals showed that a substantial, but partly latent reservoir of research capacity existed (Fig. 4). In total, 304 Letters of Intent were submitted by 242 different lead applicants in response to the five calls for proposals. Only 5% of the 242 applicants submitted a Letter of Intent in the first year and 19% in the second year, and 71 eventually led a study.

When we asked those who submitted a research proposal about their involvement in research, several of them said that they did not consider themselves to be a ‘researcher’, as they worked primarily in a different role, such as policymaker or district health director. Many applicants had heard from others about the opportunities that the research programme offered and became involved because there was funding available to study issues that were related to their own concerns, experiences and aims in the health sector, such as improving vaccination coverage, adherence to tuberculosis treatment or managing health professionals.

#### Supporting research and strengthening capacities

The programme management experimented with different approaches to monitor the quality of research and support research teams. In the first years of the programme, two research coordinators travelled throughout the country to monitor the ongoing studies and provide hands-on support. Due to the growing number of studies, this support on location became too time-consuming. To increase efficiency, newly funded researchers were invited to an orientation meeting in which they were briefed about the programme guidelines. Researchers were asked to present their work at a mid-term review and, if further support was needed, teams could request technical advice from experienced researchers from their own area.

To strengthen research capacities, the programme organised workshops that focussed on specific skills, such as qualitative data analysis and report writing. In the first years, these workshops did not seem efficient, because the number of researchers was small and they had very different needs. A programme coordinator who ran the workshops explained: “*it varies a lot* […] *we had qualitative people that could barely design tables, and those highly technical who could not bring it down to a practical level*”*.* While the increasing number of researchers in the subsequent years made the workshop strategy more successful, it remained difficult to engage the most influential health sector professionals in the workshops. “*We have tried a lot, but we haven’t been able to solve it. You want the key people who can conceptualise, write up and follow through until the presentation. Those key people are busy*”.

#### The functioning of the research projects

Interviews with the research teams provided some insight into how the programme contributed to the functioning of health research in Ghana. Participants consistently described that, without the HRDP, their studies would not have been conducted because there was no other source of funding. An investigator explained “*it is the only funding source available* […] *there are no alternative sources for doing our own research, so we rely on the programme*”*.*

Besides funding research, the research programme also helped to strengthen local research capacities. For about half of the principal investigators, it was the first time they had initiated and led their own funded research project. These investigators often emphasised how beneficial it was to them to formulate and lead their own study and be responsible for the results. “*I have gained a lot of skills from being in the lead. Writing a proposal and doing the reports, it has so much improved me*”.

Several of these researchers said that leading their own study had helped them to build their confidence and inspired them to pursue a career in research: “*I would not have moved into research. It is the first well-funded project I had. I would have put my ambition on the shelf. The research centre that I am building right now all started with this project*”.

More experienced investigators described that the programme allowed them to study issues that they had long cared about. A regional health director provides an example: “*The quality of the staff at the sub-district level is something that has bothered me for a long time and this enabled me to do some research on it.* […] *The fact that I am able to come up with a research report makes me stronger in the discussions when I raise these issues.*”

An important challenge for these more experienced investigators was finding the time to conduct their research. Many of these investigators had influential functions or advisory roles in the health sector. Their experience and networks helped them to link research to needs, but their busy agendas made it difficult for them to allocate the time for research and programme activities. Several of these investigators described that they tried to engage more junior researchers in their studies and build teams that could support them in future projects. “*We still have some of them working with us as a result of the training they received for this study*”.

In addition to strengthening the capacity of individuals, participants said that the research programme contributed to the emergence of a more conducive research environment in Ghana. “*Health research is getting bigger. More people are involved and there seems to be an emerging research culture*”. Another investigator confirmed this: “*It is helping to involve people in research and helping to keep some people in Ghana*”.

The involvement of researchers from the Netherlands was less than anticipated. Ghanaian researchers could invite Dutch researchers to collaborate with them. These Dutch researchers were funded from a different budget. Even though most Ghanaian investigators said that they liked the idea of international collaboration, Dutch researchers were involved in only 14 of the 79 funded studies. Several Ghanaian investigators said that it was not necessary to bring in a foreign researcher for their specific study. Others said it was difficult to find a partner. When we asked why they engaged Dutch researchers in their proposals, Ghanaian participants said that they hoped to benefit from specific technical expertise, sharing of experience and perhaps new opportunities for future research through international collaboration. While some Dutch researchers were happy to play a supportive role in the Ghanaian-led studies, others said that they were not very interested because they did not like the more supportive role and could not do the research that they were most interested in.

#### External review of programme performance

An external review of the HRDP in 2005 confirmed that the programme succeeded in its aim of generating and supporting demand-driven and locally led research. The review was requested by the Joint Programme Committee, who asked independent reviewers to assess whether the programme had achieved its objectives and suggest how the results could be sustained into the future. The review team concluded that, in Ghana, a well-functioning set of institutions had been developed for setting a national research agenda, generating and selecting research proposals, and supporting research on location. In the report, the research agenda was described as “*inclusive and consistent with the formal health sector priorities*”. The system for short-listing Letters of Intent and reviewing proposals was described as “*effective and highly commendable*” and the organisational structure, relations and procedures for assessing and supporting research were described as well functioning. The reviewers concluded that, overall, the HRDP had generated a research cycle that was not only demand-driven but had actively involved the Ghanaian community. Mutual trust, respect and transparency had been developed between the Ghanaian and Dutch partners and provided a solid foundation for the future.

#### Assessing the use of research

Besides the functioning of the programme, the Joint Programme Committee was also interested in whether the programme strategy increased the likelihood that results were used. In March 2005, Dutch researchers were asked to start mapping the use and impact of the funded research. This first assessment was to include all 16 studies of which a final report had been submitted to the secretariat, and should focus on whether and how the results were translated into action and explore how this related to the strategy of the research programme.

The impact assessment revealed that, within 12 months after finalisation, the results of 10 of the 16 studies had been used to contribute to action [30]. The mapping study provides some insight into the kind of studies that were funded and the way that results were used. Some studies contributed to the functioning of existing health programmes. One example is a study that assessed the quality of the immunisation programme at the district level. The study showed several shortcomings in the vaccination process and a lower than reported coverage. Several recommendations were formulated and implemented, such as a new strategy for communicating with communities, better reporting and supervision, and a policy to abolish the illegal sale of medicine and food products by health workers at vaccination sites, which prevented the poorest of the poor from having their children vaccinated. A second study showed that essential medicine and consumables needed for preventing maternal mortality were often not available in rural clinics in northern Ghana. Participants described how the results were used to improve the distribution and supplies of consumables and strengthen the documentation system. A third study showed that distance to the clinic and the costs of transportation were important reasons why tuberculosis patients did not finish their treatment [37]. The results were used to open five new tuberculosis treatment spots and decide where they should be located.

Research also contributed to the development and implementation of new health programmes. One study focussed on ways to improve quality of care in health districts. The results were used to establish indicators and quality teams for monitoring and improving quality at the district level. Another study assessed how the new Community-based Health Services and Planning Initiative could be implemented. The results were used to develop a support package for implementing this planning initiative, which was used in districts throughout the country [38].

Three studies contributed to the design and implementation of the National Health Insurance Scheme, which was a key priority of the Ghanaian government [39]. One study had shown that the poorest community members were less likely to participate in district health insurance than others and were difficult to identify [40]. The results were used to adapt a method for identifying the poor and improve the local implementation of the insurance. A second study had focussed on the perception of, and the need for, community health insurance in northern Ghana. The results were used to identify structures for collecting premiums and organise a targeted campaign to increase participation in urban districts.

A challenging question was whether the use of research was related to the demand-driven strategy of the research programme. The systematic analysis of research and translation processes showed that the priority setting and proposal selection process led to the funding of studies which were from the outset closely aligned with health sector priorities. What seemed even more important, in terms of the eventual use of the results, was that research was initiated and conducted by people who aligned research to local needs and circumstances and tried to play a role in translating results into action [30].

Between 2001 and 2005 the research programme was thus increasingly successful in generating, funding and supporting demand-driven and locally led research. During these years, there was little attention for the sponsorship constellation that supported the programme. The approval and funding of the 5-Year Programme of Work by the Netherlands government in 2001 provided, at least temporarily, a protected space that allowed those involved to focus on the functioning of the research programme and the actual research projects. The external programme review and impact assessment showed that the HRDP succeeded in generating and supporting demand-driven and locally embedded research, of which the results were translated into action.

### 2006–2008: collapse of the sponsorship constellation

In early 2005, changes in the sponsorship constellation of the HRDP started to create uncertainty about its future. The first 5-Year Programme of Work would end in June 2006 and the expectation had always been that the Netherlands government would fund another 5-year period.

A number of changes heralded the breakdown of the sponsorship constellation that supported the research programme. In the Netherlands, a new Minister for Development Cooperation had been appointed who was less interested in research and disbanded the RAWOO, which had always supported the HRDP. A second change was that decision-making about development programmes was decentralised from the Ministry of Foreign Affairs in the Netherlands to the local embassies in recipient countries. In addition, the official at the Dutch embassy in Ghana, with whom the programme secretariat had always interacted, was replaced by someone else.

The new embassy official was initially very critical of the HRDP. The new official was unfamiliar with the RAWOO and had little knowledge about the origin and functioning of the research programme. In an interview about the programme, the new official started out with arguing that health research in countries such as Ghana was much too oriented towards international scientific publications, instead of local needs and contributing to action. Soon after, the new official announced that the embassy would not continue to fund the HRDP in its current format because it had to focus on Ghana itself and did not consider the funding of a North–South research collaboration as part of its mission.

While the future of the HRDP was uncertain and no new call for proposals was permitted, the programme was allowed to use the remaining budget to continue to support the ongoing research cycles. The 31 studies that were selected for funding in 2005 started in 2006. Research teams were invited to an orientation workshop, received targeted on-site support and could participate in workshops for data analysis and report writing and final reports were printed and disseminated.

In September 2008, the curtain finally fell on the HRDP. A 2-day dissemination meeting was held in the capital Accra. The programme management invited journalists to cover the event and asked the Ghanaian Minister of Health to speak about health research in Ghana. The Dutch evaluation team that had continued to assess the use of research was invited to present their results and an official from the Dutch embassy would explain its decision about the financial support for the research programme.

The 2-day meeting showed that the HRDP had helped to further develop the Ghanaian research community and strengthen the role of health research in Ghana. The meeting was attended by nearly 200 participants and over 40 studies were presented and discussed by researchers, policymakers and other research stakeholders. In his speech, the Minister of Health emphasised the importance of health research in Ghana and leading national newspapers covered the event. Participants at the meeting described how, during the past years, the perception of research within the health sector had changed. A policymaker told how research was increasingly valued within the Ministry of Health: “*People start to recognise that research is critical*”. A director of the Ghana Health Service, who was interviewed at the meeting, described something similar: “*It is making a difference, because it is there, now there is a focus. You now see a group of people who put appreciation and a premium to research. So already we are beginning to see a research culture, a growing idea that research is relevant to the system. Without this programme this would not be there. People are interested in PhDs and the HRU* [Health Research Unit] *has got a very positive image*”.

The assessment of the use of research provided further evidence of the success of the research programme. Within 12 months after their finalisation, the results of 20 of the 30 assessed studies were translated into action [30]. Compared to other research programmes, this number seemed high. Analysis of how and why research had been used suggested that the programme strategy, with its emphasis on demand-driven and locally led research, was an important factor behind this success rate.

While the new official at the Netherlands embassy had become more positive about the research programme and recognised its success, he still announced that the Netherlands government would end the direct funding of the HRDP. The new official described the 2005 Paris declaration on Aid Effectiveness as the main reason for not continuing the direct funding. Central to the Paris declaration was the commitment to help the governments of developing countries formulate and implement their own national development plans, according to their own national priorities, using, wherever possible, their own planning and implementation systems. Keywords were ownership, alignment and harmonisation. Aid had to be pooled in support of a particular strategy led by a recipient country – a national health plan, for example – rather than being fragmented into multiple individual projects. For the new embassy official, this meant that the HRDP should no longer be funded as a separate programme. Instead, all funding should be provided to the Ghanaian government as part of multi-donor budget support for the health sector. National priorities should determine if the money was to be allocated to health research. This decision brought an end to the formal existence of the Ghanaian-Dutch HRDP.

The official of the Netherlands embassy presented this decision as a new phase in the development of health research in Ghana. Ghanaian researchers were critical in their response to the idea that this was a new phase. They pointed out that, for years, local researchers had lobbied with the government for a reasonable budget for research. The Ministry of Health had always welcomed the idea, and even pledged to allocate 5% of the budget of the Ghana Health Service to research, but had so far not provided additional funding. At the 2008 dissemination meeting, an official of the Ministry announced that it would establish a budget line for research and was planning to play a larger role in health research. When, at the meeting, a critical researcher asked about the budget plans of the Ministry, the official admitted that it was unlikely that new funding would be allocated to research in the 2009 budget plan.

During an interview in early 2009, we asked two officials from the Ministry of Health why the Ministry had not increased its funding for research. The participants explained that, while research was seen as important, senior staff at the ministry considered research a domain for which a lot of international funding was available. “*Before the Dutch, we had the British and the Swedes, and now there is a lot of American funding, you know, USAID, Gates. There is the WHO and Global Fund and there are many others*”. The participants explained that, while funding for research seemed available, the Ministry was constantly struggling with a lack of resources and an uncertain stream of donor-driven funding and changing development trends. As a result, those in charge at the ministry had a strong preference for investing in concrete projects with clear short-term results.

Without a realistic budget for demand-driven and locally led research, the organisational arrangements that were set up to run the demand-driven research programme were not maintained. Core staff of the programme continued to lead the existing Health Research Units, secured new research grants from international and donor agencies, and moved on to new positions and other organisations.

## Discussion

The aim of this study was to analyse how a programme for demand-driven and locally led research in Ghana, which was supported by a North–South collaboration with the Netherlands, came into being and functioned in practice.

The results show how the development of the research programme started in the early 90s in the Netherlands, with the construction of a sponsorship constellation. After showing the problems with traditional research collaboration, an advisory council formulated a vision for a more equal and effective approach to North–South collaboration. Together with Ghanaian partners, this vision was turned into a proposal for a Ghanaian-led programme for demand-driven and locally led research, which was funded by the Netherlands government in 2001. Research priority setting showed that there was a true need for demand-driven research. After a slow start, the number and quality of the proposals that were submitted rapidly increased and the programme became increasingly successful in generating, funding and supporting demand-driven and locally led research.

The third part of the analysis shows how, despite the strong performance of the programme, its role in supporting locally led research and the use of the results, the research programme came to an end in 2008 because its sponsorship constellation in the Netherlands collapsed and attempts to mobilise new funding in Ghana were unsuccessful.

The struggle that emerged in the Netherlands when development funding was allocated to research that would be led by researchers from the South is remarkable, but not unique. There are several studies that show how the interests, priorities and actions of researchers from the North can constrain the development of demand-driven research in low-income countries [6, 28, 41]. For decades, researchers from the North who work in low-income countries have used development funding to do the research that they believe is needed most. After years of rising research capacity in the South, development funding can also be used to fund locally led research. This leads to competition over priorities and limited resources. While investing in Southern-led research seems more effective and efficient from a development perspective, researchers from the North continue to play a dominant role in global health research and have much better access to research and development funding [42, 43]. The demand-driven programme approach of the HRDP provides a model for North–South collaboration that increases the role of Southern researchers, strengthens local capacities and institutions, and invests in research that is aligned to local needs and likely to be used to improve local action.

### The need for demand-driven research

The Ghanaian national research agenda shows that there was a true need for demand-driven research. The local research needs, which were mostly related to health systems, differed substantially from the disease-specific, biomedical studies that foreign researchers and funders were mostly interested in. The stark disagreement between international and national research priorities shows why countries need to set their own national research agenda and provides support for those who promote priority setting worldwide [44–46].

Besides the difference in priorities, the Ghanaian research agenda also shows that there was a strong need for locally specific research. While national research agendas in countries around the world show a similar need for locally specific research, there is little attention for such research in the current discussions about research for global health [15, 27]. International agencies, funders and the scientific community tend to focus on disease-specific research that aims to produce universally valid knowledge claims [42].

While this universally oriented research can lead to useful insights and innovations, it provides, at best, a small part of the knowledge that is required for improving health in the South. The one-sided focus on producing knowledge that is intended to be universally valid and applicable leads to a neglect of more locally specific research that policymakers, health professionals and community representatives in low- and middle-income countries say they need to improve action for health. A largely neglected challenge is finding the right balance between more locally specific and more universally oriented research. When trying to find this balance, it is essential to look beyond the promise of universal applicability of research findings, and analyse the extent to which the research that is funded is actually used to contribute to action for health [31, 47].

### Mobilising the existing research capacity

An important lesson from the HRDP is that the research capacity that existed in Ghana was much larger than it seemed in the first years of the programme. Surveys of research capacity, analyses of numbers of publications and ‘calls to action’ consistently suggest that the research capacity that is present in the South is relatively limited [42, 48, 49]. The idea that research capacity is limited is used to support investments in capacity strengthening programmes and legitimise the substantial role that foreign researchers continue to play in these countries [24, 27, 28, 41].

The experience of the HRDP shows that, in Ghana, a substantial, but partly latent reservoir of professionals with relevant research skills was present. A substantial part of this reservoir does not appear in lists of publications or formal research positions because it showed itself only after a regular funding opportunity for locally led research was available. The existence of a latent reservoir of research capacity is not explicitly described in the literature about capacity strengthening, but it is not surprising. For years, researchers from low-income countries have argued that the lack of funding for locally led research is the main reason why they did not continue to work in research [6, 22, 27, 50]. While training new researchers is needed in every research system, our study shows that the key to strengthening research capacity in countries such as Ghana is to increase the funding that is available for locally led research.

To mobilise local capacity for demand-driven research, a set of well-functioning local institutions and networks is required. In Ghana, the HRDP benefitted from the support of leading policymakers in the health sector and an existing research tradition and infrastructure upon which the institutions for demand-driven research could be constructed. Despite these conducive circumstances, those who ran the programme still had to develop, test and improve new procedures, norms and rules for making the programme function as intended. As others have shown, realising well-functioning demand-driven research programmes requires a long-term perspective, careful and inclusive preparation, and sufficient resources, capacities and time for developing the required procedures, infrastructure and networks [51, 52].

An important finding is that the research that was funded was often used to contribute to action [30]. The detailed analysis of which studies were used and why showed that the use of research was related to the demand-driven approach, and especially the fact that research was initiated and led by local researchers, who aligned their research towards specific needs and local circumstances and played a role in the translation of knowledge into action. This shows that the strategy of the programme, to support demand-driven and locally led research, was working as intended.

### The essential role of a sponsorship constellation

As a final result, our study provides insight into the critical role and dynamics of sponsorship constellations. While the importance of political, societal and especially financial support for research is widely acknowledged, there are very few empirical analyses of how such support emerges, stabilises and functions, especially in low- and middle-income countries. Inspired by others, we undertook a multilevel analysis and introduced the notion of a sponsorship constellation to refer to the network of actors that mobilises funding for a research programme and legitimates its role in society [26]. The analysis provides some insight into how such a sponsorship constellation came into being, fulfilled its role and fell apart, which is perhaps also relevant for those who manage knowledge translation platforms, communities of practice and learning networks in global health [53, 54].

Our multilevel analysis shows that, while the HRDP succeeded in realising a well-functioning research programme in Ghana, it remains risky when such a programme depends on a single sponsorship constellation that is located in a foreign country. An essential and rarely studied question is how local sponsorship constellations for demand-driven research programmes can be established in low- and middle-income countries. On several occasions, ministers of health from African countries and elsewhere have pledged to allocate at least 2% of their national health budgets to health research [55, 56]. While some countries have increased their investments in research, the sparse data that is available indicates that the funding that is allocated is nowhere near the pledged amount [42]. The description of how the sponsorship constellation for the HRDP was forged in the Netherlands may provide some indication of the time it may take and the kind of the efforts that may be required to realise such constellations.

### Considerations for research policy

Our analysis allows us to formulate some suggestions for those who aim to strengthen the functioning of research in low-income countries and elsewhere.

A first suggestion is to increase the funding for demand-driven and locally led research in low- and middle-income countries. Worldwide, over US$240 billion is spent annually on health research [42]. While only a small percentage of this seems directly relevant for health in low-income countries, this fraction is still mostly spent on disease-specific research that is prioritised internationally and initiated and led by researchers from the North. Even if only 0.1% of the global investment would be shifted towards demand-driven and locally led research in low-income countries, this would result in a fundamental change in the research landscape in these countries, an enormous boost for local capacity and a substantial increase in locally relevant research that is likely to be translated into action for health.

A second suggestion is to consider a ‘demand-driven programme approach’ as a strategy for strengthening research capacity in the South. During the past decades, a variety of approaches for research capacity strengthening have been promoted, particularly with respect to training individual researchers and developing and strengthening research centres and systems [17]. Our analysis shows the merits of developing a nationally embedded programme for demand-driven and locally led research, which all countries ultimately need [57, 58]. Some advantages of a ‘demand-driven programme approach’ are that it orients research to local needs, helps to strengthen and mobilise local capacity and institutions, and provides a clear aim for which different components most be realised.

A third suggestion is to increase the attention for realising the local sponsorship constellations that are required for demand-driven research programmes. To move beyond dependency on unpredictable foreign funders and create sustainable research programmes, it is essential to construct local sponsorship constellations that generate sufficient funding. While forging such constellations requires a locally led process, international agencies can perhaps provide some financial and technical support, by sharing lessons learned in other countries, for instance. To encourage and support governments to develop such local sponsorship constellations, international funders could offer co-funding for demand-driven research programmes [59].

### Limitations

The main limitation of this study is that we started the data collection process in 2005. To reconstruct the long preparatory process, we had to ask participants about events that happened several years prior to our first interviews. We tried to prevent recall bias by checking claims with the extensive documentation of the preparatory process, which included several reports and a detailed process description that was written by one of the participants.

## Conclusion

Our study shows that developing a programme for demand-driven and locally led research in a low-income country provides an effective approach to North–South collaboration. The research programme that was developed in Ghana generated, funded and supported demand-driven and locally led research, of which the results were often used to contribute to action. The programme helped to strengthen local research capacities, institutions and networks. More attention is needed for realising the local sponsorship constellation of research.

## Abbreviations

HRDP: Ghanaian-Dutch Health Research for Development Programme
RAWOO: Development Assistance Research Council
NWO: Netherlands Organisation for Scientific Research.
